# Laryngeal papillary carcinoma with unexpected evolution: case report

**DOI:** 10.1590/S1516-31802006000300010

**Published:** 2006-05-04

**Authors:** Adriano Santana Fonseca, Carlos Takihiro Chone, Agrício Nubiato Crespo, Albina Altemani

**Keywords:** Laryngeal neoplasms, Papillary carcinoma, Radiotherapy, Prognosis, Lymphatic metastasis, Neoplasias laríngeas, Carcinoma papilar, Radioterapia, Prognóstico, Metástase linfática

## Abstract

**CONTEXT:**

According to the literature, laryngeal papillary carcinoma is rare and has a benign prognosis.

**CASE REPORT:**

In this report we present a surprising case with nodal metastasis at the time of diagnosis. Computed tomography showed infiltration of the lesion and metastatic lymph nodes. The resected specimen was submitted to histopathological study that confirmed the diagnosis of papillary squamous cell carcinoma.

## RESUMO

**CONTEXTO:**

Segundo a literatura, o carcinoma pilífero de laringe é raro e tem um bom prognóstico, quando comparado aos outros carcinomas espinocelulares.

**RELATO DE CASO:**

Neste relato, apresentamos um caso já avançado ao diagnóstico, com acometimento linfonodal e invasão extracapsular bilateral. Tomografia computadorizada mostrou infiltração da lesão e metástase em linfonodos. Após cirurgia, o espécime ressecado foi submetido a estudo histopatológico, que confirmou o diagnóstico de carcinoma papilífero.

## INTRODUCTION

Papillary squamous cell carcinoma is an entity characterized by highly atypical squamous cell proliferation. Stromal invasion, however, is rarely seen.^1,2^

The evaluation of an exophytic or papillary lesion should always include differential diagnosis in relation to other lesions presenting the same growth pattern, such as verrucous carcinoma, but without the high count of atypical cells found in papillary squamous cell carcinoma. Squamous papillomatosis may present with a high count of atypical cells,^1^ but also with a high count of koilocytic cells,^1^ and should be differentiated from papillary squamous cell carcinoma. The importance of the differential diagnosis lies in the different prognoses for these lesions.

Recent studies have demonstrated better prognosis for papillary squamous cell carcinoma with a papillary pattern than for papillary squamous cell carcinoma with the exophytic pattern or for conventional squamous cell carcinoma.^2^ On this basis, a modification to the therapy for this kind of tumor has been put forward, with regard to its aggressiveness.^2^

In this report, the authors present a case of papillary squamous cell carcinoma with papillary patterns that had an unusual malignant evolution, for which radical surgery, adjuvant chemotherapy and radiotherapy were used in the treatment.

## Case report

A 42-year-old white female was referred to our clinic with a complaint of dysphonia and pain in the right cervical area. The patient had no complaints of dyspnea, dysphagia or odinophagia. She had no previous history of tobacco or alcohol use, and no symptoms related to gastroesophageal reflux.

Fiberoptic examination of the upper airway was carried out and this showed an exophytic verrucous lesion occupying the posterior third of the left vocal and vestibular fold. It had a submucous and infiltrative pattern, and there was decreased mobility of the left vocal fold. A lymphatic metastasis of 5.0 cm was found at the right level IV, featuring hard consistency and adherence to the deep tissues. The lesion was clinically staged as laryngeal, cT2N2cM0. Biopsy was performed on the posterior laryngeal area and fine-needle aspiration on the enlarged cervical mass. The specimen was also sent for hybridization *in situ* and electron microscopy.

The histopathological findings from the laryngeal specimen indicated well-differentiated squamous cell carcinoma. Fine-needle aspiration biopsy on the right cervical mass showed atypical squamous cells compatible with squamous cell carcinoma, upon histo-pathological evaluation.

**Figure 1:**
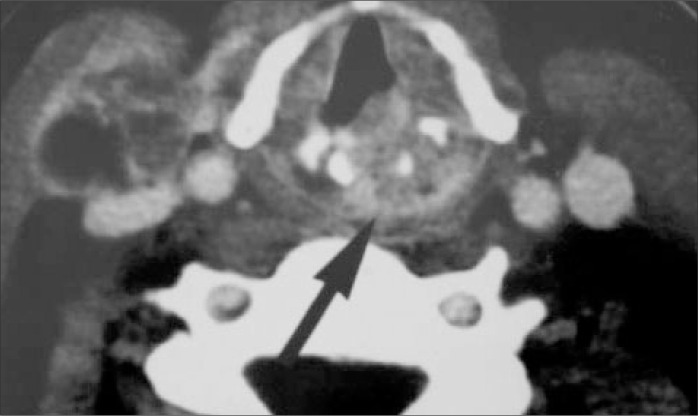
Axial computed tomography scan with lesion infiltrating posterior half of vocal fold, arytenoid cartilage and surrounding tissues (black arrow). Note the lymph node metastasis at level IV (right side).

The computed tomography scan showed that the lesion had infiltrated the posterior third of the vocal fold, left arytenoid cartilage and interarytenoid portion and destroyed the upper posterior half of the cricoid cartilage. The lesion was also occupying the left paraglottic space. In addition, images of metastatic lymph nodes at left level II and right level IV were noticed (Figure 1).

The patient underwent total laryngectomy with bilateral modified radical neck dissection and right anterior compartment neck dissection. A primary tracheoesophageal puncture for voice rehabilitation was also performed.

**Figure 2:**
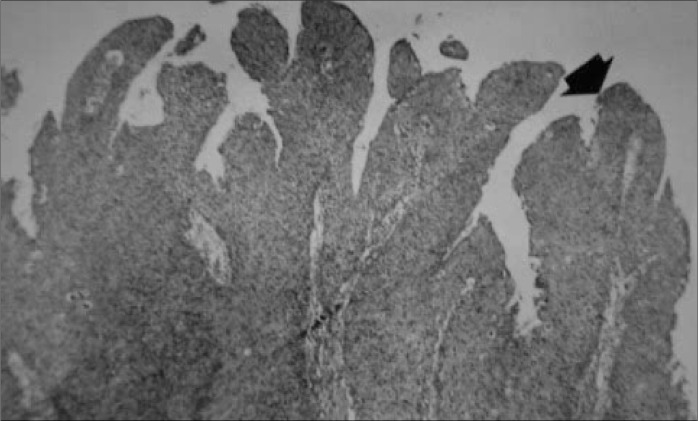
Black arrow shows fingerlike projections of the papillary pattern of papillary squamous cell carcinoma, in histopathological study of surgical specimen resected from the larynge of a 42-year-old woman.

The evaluation of the surgical specimen using histopathological study confirmed the diagnosis of papillary squamous cell carcinoma with a papillary pattern (Figure 2), involving the posterior half of the left vocal and vestibular fold, the posterior half of the left ventricle, and the left arytenoid cartilage. There was one positive lymph node on the right side at level IV, measuring 4 x 2.8 cm in size with extracapsular spreading. On the left side, one lymph node demonstrated metastatic disease at level II, measuring 2.8 x 2.3 cm, also with extracapsular spreading.

The patient underwent adjuvant treatment using chemotherapy, consisting of three cycles of 5-fluoracil and cisplatin, and radio-therapy with a total dose of 5000 cGy.

The hybridization *in situ* revealed no evidence of human papillomavirus in any of the specimens obtained.

## DISCUSSION

Papillary squamous cell carcinoma is a variant of squamous cell carcinoma. The latter accounts for up to 90% of all the malignant lesions found in the larynx.^3^

The histological variants of squamous cell carcinoma include verrucous carcinoma, sarcomatoid carcinoma, basaloid carcinoma, adenocarcinoma and cystic adenoid carcinoma.^2,4^ These may also be divided into papillary or exophytic types, according to their growth pattern. The exophytic pattern presents rounded and cauliflower-like projections, whereas the papillary pattern resembles more delicate fingerlike projections. The classification as exophytic or papillary should follow the dominant pattern that corresponds to at least 70% of the tumor size.^2^ The diagnosis of papillary carcinoma is made through histological examination when there is a high count of atypical cells and low rate of koilocytosis.

## Conclusion

In our report we have described a case of papillary squamous cell carcinoma in a young patient, at an unusual site in the larynx, with positive lymph nodes and extracapsular spreading. The observed outcome was the opposite of what is presented in the current literature, which describes a good prognosis for this lesion.^1–5^
